# Fabrication and Coating of Porous Ti6Al4V Structures for Application in PEM Fuel Cell and Electrolyzer Technologies

**DOI:** 10.3390/ma17246253

**Published:** 2024-12-21

**Authors:** Juan Villemur, Carlos Romero, Jose Manuel Crego, Elena Gordo

**Affiliations:** 1 Department of Material Science and Engineering, Universidad Carlos III de Madrid, IAAB, 28911 Leganés, Madrid, Spain; 2 Department of Applied Mathematics, Materials Science and Engineering and Electronic Technology, Universidad Rey Juan Carlos, 28933 Mostoles, Madrid, Spain; carlos.romero@urjc.es

**Keywords:** Ti64, porous, titanium nitride, corrosion, interfacial contact resistance, PEM

## Abstract

The production of green hydrogen through proton exchange membrane water electrolysis (PEMWE) is a promising technology for industry decarbonization, outperforming alkaline water electrolysis (AWE). However, PEMWE requires significant investment, which can be mitigated through material and design advancements. Components like bipolar porous plates (BPPs) and porous transport films (PTFs) contribute substantially to costs and performance. BPPs necessitate properties like corrosion resistance, electrical conductivity, and mechanical integrity. Titanium, commonly used for BPPs, forms a passivating oxide layer, reducing efficiency. Effective coatings are crucial to address this issue, requiring conductivity and improved corrosion resistance. In this study, porous Ti64 structures were fabricated via powder technology, treating them with thermochemical nitriding. The resulting structures with controlled porosity exhibited enhanced corrosion resistance and electrical conductivity. Analysis through scanning electron microscopy (FE-SEM), X-ray diffraction (XRD), grazing incidence XRD and X-ray photoelectron spectroscopy (XPS) confirmed the effectiveness of the coating, meeting performance requirements for BPPs.

## 1. Introduction

In recent decades, interest in eco-friendly and highly energy-efficient systems has grown exponentially, with hydrogen technologies being one of their prime examples [1]. Hydrogen can be used as a substitute in virtually all applications where fossil fuels are currently used, while eliminating pollutant emissions [2,3]. According to H_2_ Council forecasts, it is estimated that hydrogen will account for 18% of global energy consumption by 2050 [4]. Therefore, the development of green hydrogen technology is crucial for achieving a ’hydrogen society’ where energy from less stable renewable sources can be stored in various forms of hydrogen, whether compressed, liquefied, or in hydrides [5]. There are many types of hydrogen, but three are the primary ones: gray, in which hydrogen is obtained from natural gas, emitting CO_2_ into the atmosphere; blue, in which it is also produced from natural gas, but with the implementation of capture, utilization, and storage technologies for CO_2_ emissions; and green, in which hydrogen is obtained from electricity generated from renewable energy sources and water, emitting O_2_ into the atmosphere. The production of green hydrogen is the most expensive of these [6], and therefore, research is needed to develop cheaper or more efficient technologies, as only 4% of the total hydrogen produced comes from water electrolysis [7], while 63% comes from gray hydrogen [8].

Currently, alkaline water electrolysis (AWE) and proton exchange membrane water electrolysis (PEMWE) are widely used for the production of green hydrogen [1,9,10], accounting in 2022 for 59% and 32% of the global electrolysis capacity, respectively. Although AWE is the most widely used, it has a number of disadvantages that limit its use, such as a low charge range, large stack size, and limited current density [1,9]. Therefore, PEMWE technology represents an interesting alternative as it can work with higher current densities and a wider power range. One of the advantages of this technology is the small thickness of the membrane that allows the protons to migrate a very short distance, reducing the ohmic losses. In addition, the catalyst on the membrane surface easily transfers the protons from the reaction zones to the solid electrolyte, considerably reducing the limitations due to mass transport [9]. The cell efficiency can be expressed as a function of the real (U_cell_) and theoretical (E) operating potentials according to Equation (1), these potentials being dependent on temperature, pressure, and current density.

(1) $$\varepsilon_{∆G}\left(T,P,j\right)=\frac{E(T,P)}{U_{cell}(T,P,j)}$$

At low intensities, efficiencies close to 100% are obtained, according to this equation. Efficiency is a crucial parameter responsible for the energy cost of the process. The use of high current allows the initial investment to be reduced at the expense of efficiency, so it is important to find a compromise between cost and efficiency, reducing the use of noble metals in its components [11].

One of the main components of a PEM electrolyzer is the bipolar plates (BPPs). Their objectives are the distribution of gases in the cells, electron transfer, heat transfer, mechanical integrity of the cell, and distributing the reactive agents inside the electrolyzer [12,13]. Due to the harsh operating conditions reached in the electrolyzer (1.6–2 V, high temperatures between 65 °C and 80 °C, and acidic conditions between pH 2 and 4), there are few materials that can perform this function in long-term operations [1]. Added to this is the need to reduce the cost of the electrolyzer, wherein the BPP accounts for between 30% and 40% of the total cost [14,15]. Thus, the BPP must have high corrosion resistance, high mechanical strength, high durability, and easy manufacturability for both mass production and cost reduction. Several studies have been conducted on the manufacturing, structure, pore size and distribution, and composition of BPPs, researching to produce protective coatings and suitable materials.

Metallic BPPs have been chosen due to their better mechanical resistance, which allows them to be machined and withstand the forces exerted for the assembly of the cell, as well as their greater ease of mass production compared with other BPPs made from graphite or polymers, thus allowing them to be reduced in size and achieve a reduction in both the weight and size of the PEMWE [16]. In addition, both carbon structures and carbon coatings have been used in the PEMFC on the anode and cathode side. However, due to the operating conditions present at the anode of a PEMWE cell, their high oxidation potential as well as their low mechanical strength do not allow the use of these materials [9]. However, metallic BPPs have two clear disadvantages compared with graphite BPPs. Firstly, the electrical conductivity of graphite is higher than that of many of the metals used to obtain bipolar plates. Secondly, there is corrosion [17]. This factor affects both the contamination of the membrane with metal oxides from the BPP and the electrical conductivity of the plate itself [18]. The vast majority of metals develop passive oxide layers in response to corrosion, which have an insulating character.

Many studies have been carried out on different metallic materials for application in BPPs. Stainless steels have been some of the most studied, due to their low cost, easy machinability, and good corrosion resistance. Li et al. [19] studied the electrochemical behavior of 316L BPPs coated with Ta, obtaining a low corrosion density (I_corr_) of 1.4 × 10^−7^ A·cm^−2^. Yoon et al. [20] investigated the influence of a Zr coating on an AISI 316L, obtaining low values of current density. However, this material presents surface oxidation, which increases the interface contact resistance (ICR) and causes membrane poisoning: it corrodes, releasing Fe and Cr ions that are deposited on the membrane and catalysts, degrading them and worsening the performance of the PEMWE [21,22]. This leads to the need for research into coatings [23], usually of graphite or precious metals, which improve the substrate’s resistance but, in turn, represent an increase in the cost of the component [24,25].

Titanium is presented as a strong alternative as it has a good specific strength, allowing a decrease in the total weight of the BPP, as well as excellent corrosion resistance without the need to apply any type of coating to the surface. Cheng et al. [26] optimized the corrosion resistance of additive manufacturing Ti6Al4V by heat treatment process, obtaining a more stable oxide layer and enhancing corrosion resistance. However, titanium has a high cost, both in terms of raw material and formability, which considerably limits its use [27]. In terms of machinability, obtaining titanium BPPs presents complications in obtaining small channel sizes and thicknesses, since it has a low degree of deformation prior to fracture. In addition, their weldability is limited due to their low thickness, presenting a risk of rupture. Apart from the search for a way of processing that improves both the cost and the possibility of mass production, research is also focused on the development of coatings that improve the properties of the substrates, especially avoiding the insulating Ti oxide layer formed on the surface.

As mentioned above, the main coatings used are those based on precious metals such as platinum or gold [28], but due to their high cost, other types of coatings have started to be used, such as graphitic, polymeric, or metallic coatings. For example, Wakayama et al. developed a NiTiP coating [29], and Gao et al. used titanium BPPs with carbon/PTFE/TiN composite coatings [30]. However, coatings using nitrogen compounds are of great interest, as they form compounds that increase corrosion resistance while maintaining low electrical resistance. Sun et al. [31] developed a highly conductive and corrosion-resistant NbN coating, which exhibited low corrosion current density (1.1 × 10^−8^ A·cm^−2^) and a low ICR value (15.8 mΩ·cm^2^). For this reason, a lot of research has been carried out in order to obtain a coating with nitrides, mainly TiN [1,32,33,34]. There are different processes for the deposition of TiN coatings [35]; however, since we have a titanium substrate, treatments such as nitriding or electrochemical nitriding would be sufficient since we have to incorporate nitrogen on the surface of the titanium substrate only. These processes are simpler and do not require geometry with specific requirements, thus reducing the cost of the BPP.

One of the parameters that most affects the performance of the PEMWE is the design of the bipolar plates, as it affects the homogeneous distribution of the reactive gases on the catalyst surface and the area of the BPP through the flow channels [36]. Bipolar plates with flow channels have a number of disadvantages such as pressure losses, high manufacturing cost, and low mechanical strength, which increases the size and weight of the BPP in addition to a possible uneven flow distribution, resulting in a preferential utilization of an area of the catalyst [37]. Therefore, research is being carried out on the replacement of the flow channels by porous structures that can significantly improve the performance of both the BPPs and the BPP+PTL (‘Porous Transport Layer’) assembly. The fabrication of structures that present a porosity gradient through these components can favor the processes that take place both in the transport of gases and on the catalyst surface [38]. To obtain these advanced designs, powder metallurgy allows the fabrication of different structures with the desired porosity gradient as well as the possibility of adapting the process for the mass production of the structures from different materials. The use of space holders provides us with a very versatile route for obtaining structures with different types and sizes of porosity and, in the case of titanium, it is the most efficient technology for obtaining porous structures [39].

In this article, the effect of porosity in Ti64 samples obtained from the conventional powder metallurgical route (pressing and sintering) was studied by using space holders for their use as bipolar plates. Likewise, surface modifications were performed by nitriding to obtain TiN layers on the surface. The performance of these materials was studied using the targets set by the US Department of Energy for PEMFC BP as reference.

## 2. Materials and Methods

### 2.1. Processing of Substrates and Surface Modification

**Figure 1:**
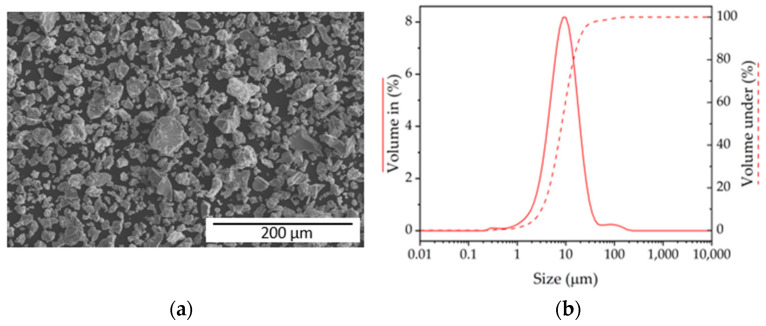
Characteristics of Ti-6Al-4V powder: (**a**) SEM micrograph and (**b**) particle size distribution.

The dense and porous Ti-6Al-4V specimens were processed via PM routes, including the space holder technique, to obtain the porous structures. The materials were processed using a commercial blend of Ti-6Al-4V powder (PARAMEET INT’L, CO, Seoul, Republic of Korea), which combined irregular Ti powder with Al powder and Al-V master alloy (Figure 1a). The powder blend had a maximum particle size of 80 μm (Figure 1b). To obtain the porous samples, ammonium bicarbonate (NH_4_HCO_3_, Sigma Aldrich BioUltra, purity ≥ 99.5%, St. Louis, MO, USA) was used as a space holder.

Dense samples were obtained directly from the Ti-6Al-4V powders, pressed uniaxially into disks of 16 mm diameter and 1 mm height using a compaction pressure of 200 MPa. For the porous specimens, the Ti-6Al-4V powders were mixed with sieved NH_4_HCO_3_ particles (30% by weight) to obtain the expected porosity with sizes up to 150 µm. Mixing was performed during 1.5 h at 50 rpm, and the mixture was pressed uniaxially under 200 MPa. Later, the space holder was removed and the samples left in a muffle at 50 °C for 8 h.

Both dense and porous specimens were sintered in two different atmospheres: in high vacuum of 10^−5^ mbar or in an atmosphere of Ar. In both cases, the samples were sintered at 1200 °C for 2 h, using a heating curve of 5 °C/min from room temperature and cooling at 5 °C/min until room temperature.

Surface modification was performed on the specimens, both dense and porous, using gas nitriding to develop layers of TiN on the substrates. The samples were heated to 1000 °C at a heating rate of 5 °C/min in a furnace under an atmosphere of N_2_. The temperature was held for 2 h.

For both sintering in Ar and surface modification, a purge of the chamber was performed during 1 h using Ar (for the sinter process) or N_2_ (for the nitriding cycle) to ensure a homogeneous atmosphere.

### 2.2. Microstructural Analysis and Density

To obtain the porosity of the samples, the density was measured using two techniques: (i) measuring the dimensions and mass of the samples to calculate the “geometrical density”; this value provides the total porosity of the sample considering the theoretical density of the alloy (Equation (2)); (ii) using helium pycnometry (Ultrapyc 5000, Anton Paar, Graz, Austria), which provides ρ_pycnometer_, and the closed porosity of the sample can be obtained using Equation (3). The difference between the total porosity and the closed porosity will be the open porosity (Equation (4)), which should be provided by the space holder. In this case, ρ_bulk_ is the bulk density of Ti-6Al-4V, 4.42 g/cm^3^.

(2) $$Total\;porosity\;\left(\%\right)=100-\frac{\left(\frac{m_{sample}}{V_{sample}}\right)}{\rho_{bulk}}\cdot 100$$

(3) $$Closed\;porosity\;\left(\%\right)=100-\frac{\rho_{pycnometer}}{\rho_{bulk}}\;$$

(4) $$Open\;porosity\;\left(\%\right)=Total\;porosity\;\left(\%\right)-Closed\;Porosity\;\left(\%\right)$$

In addition to these calculations, porosity can be examined by image analysis to determine the shape, size, and distribution of the pores. ImageJ 1.54g software was used to process 5 images of the Ti64 porous structures. The pore diameter is calculated by the software from binary images where the porous and dense zones are separated. It counts the pixels of the porous zone that are connected to each other, providing the total area they represent.

Scanning electron microscopy (SEM) using an FEI Teneo equipped (Hillsboro, OR, USA) with microanalysis probe (EDS) was performed to analyze the morphology of the produced coated samples. The working distance and accelerating potential were between 10.0 and 10.3 mm and 15 kV, respectively. The composition of the TiN coating was determined by energy-dispersive X-ray spectroscopy (EDS), with the same conditions of working distance and accelerating potential.

### 2.3. Surface Composition and Structure

The composition and crystal structure of the surfaces of treated and untreated Ti-6Al-4V specimens was characterized using X-ray diffraction (XRD), grazing incidence XRD (GIXRD), and X-ray photoelectron spectroscopy (XPS). Conventional XRD was used in an X‘Pert MPD diffractometer, with a Bragg–Brentano configuration and a Cu anode as X-ray source, to analyze the crystal structure of the surface of the material up to hundreds of microns of depth. The range studied was between 20° and 80°, step size of 0.02° and 2 s per step. GIXRD was performed on an X’Pert MRD diffractometer with a grazing incidence of 1.5°, which has a penetration depth of around 0.75 µm. XPS was used to measure the composition and the chemical state of the surface of the specimens. An Al X-ray source was used in a Fisons Instrument VG Microtech MT-500 spectrometer on as-received surfaces (Glasgow, UK).

### 2.4. Corrosion Testing

Corrosion tests were performed using a three-electrode cell, with a platinized-Ti counter electrode and a Ag/AgCl reference electrode in a solution of H_2_SO_4_ with pH = 3 at 70 °C, simulating the conditions found in the anode and in the cathode side of the BP of a PEMFC. For the anodic conditions, the test consisted of a polarization curve between −0.4 and 0.6 V vs. Ag/AgCl with Ar purge, while the cathodic conditions were evaluated using chronoamperometry for 24 h at 0.6 V vs. Ag/AgCl with air bubbling.

### 2.5. Interfacial Contact Resistance

Interfacial contact resistance (ICR) was measured using carbon gas diffusion layer (SIGRACET GDL 28 BCE), a DC power supply using 2 A of current, Au-coated copper plates, and a micromechanical testing machine (Microtest, Madrid, Spain) that can apply up to 5 kN of load. Measurements were taken according to Wang’s method [40], performed by measuring first the voltage drop of a wafer of GDL with the same contact area as the sample for different loads (V_1_) and measuring the voltage drop of the sample sandwiched in between the GDLs (V_2_). The ICR was calculated following Equation (5), where I is the current and A is the cross-sectional area of the specimen:

(5) $$ICR=\frac{V_{2}-V_{1}}{I}\frac{A}{2}$$

Pristine samples with surface treatments were measured as obtained, while untreated samples were tested after grinding using SiC paper up to 1200 grit size. ICR was also performed on specimens after corrosion testing.

## 3. Results

### 3.1. Characterization of Density and Microstructure of Porous Ti-6Al-4V

The density of the titanium structures is shown in Table 1. The total porosity is lower than the volume of space holder incorporated in the mixture (55% vol). This decrease in the porosity obtained is probably due to the shrinkage of the material by about 30% during sintering, which results in an increase in the closed porosity by trapping some of the smaller open porosity that may remain in the sample and decreasing the open pores left by the space holder. Furthermore, it must be noted that the size of the space holder is sieved to be lower than 150 µm, but the minimum particle size is not controlled. This means that some of the smaller space holder particles may leave spaces small enough to be reduced during sintering.

**Figure 2:**
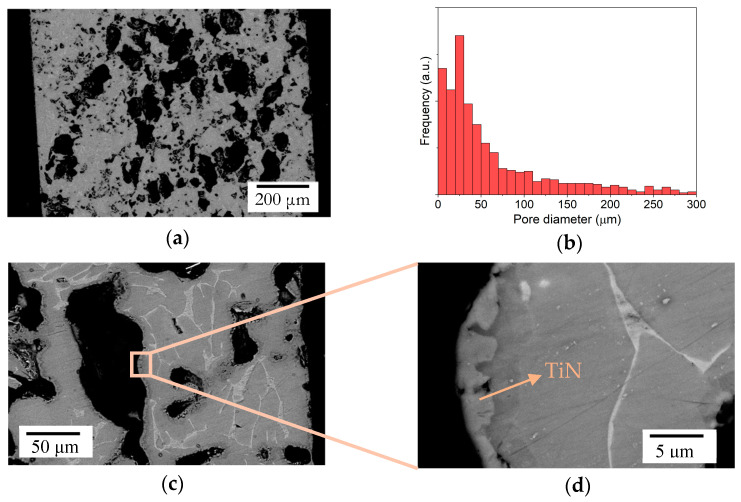
Scanning electron microscopy (SEM) images of porous Ti-6Al-4V specimens: (**a**) cross-section of a Ti-6Al-4V porous sample, (**b**) pore size distribution, (**c**) inner pore in a Ti-6Al-4V porous sample, (**d**) surface of an inner pore.

Figure 2a shows a porous Ti-6Al-4V material. The morphology of the pores left by the space holder is irregular, as the NH_4_HCO_3_ particles typically have angular, irregular shapes. The pore size is variable (Figure 2b), close to being bimodal, where the fraction of pores due to pores smaller than 30 μm represents about 40% of the total. This is due to the presence of pores left by the smallest space holder particles and those that come from the sintering of the alloy. However, there is a predominance of larger pores due to the large amount of space holders introduced, which, because of the coalescence of the particles, formed pores with sizes bigger than 150 μm. This phenomenon led to the interconnection of the pores, as evidenced by the results seen in Figure 2c,d, where the coating is obtained on the surface of an inner pore. This is the aim for the type of component to be used, and it also fits well with the surface modification used in this work, as it depends only on the migration of N_2_ molecules to the interior of the porous structure to be able to form a nitrided surface.

### 3.2. Surface Modification

**Figure 3:**
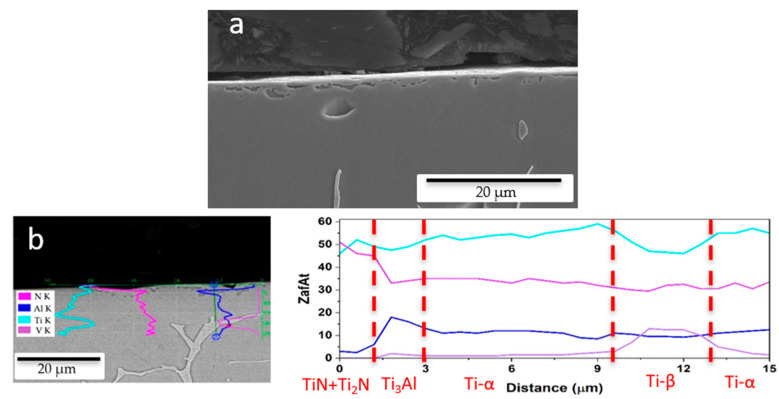
Scanning electron microscopy (SEM) images of dense Ti-6Al-4V specimens: (**a**) cross-section of a Ti-6Al-4V dense specimen, (**b**) EDS analysis of the surface of the specimen.

The SEM micrographs in Figure 3 show the structure of the Ti-6Al-4V alloy after the nitriding at 1000 °C for 2 h. The surface has a layered structure due to the varying amounts of N in solution, as is typical in a diffusion-based process. The N-rich compounds are formed on the surface as a layer of 1–2 microns, which is compact and continuous. Within 5 microns in depth, a transition region is formed, where the N concentration is lower but still high enough to promote the formation of a solid solution of α-Ti rich in N. Deeper in the specimen, the N content is low, almost negligible, and the typical α + β microstructure of Ti-6Al-4V is found. This structure is also found in the porous Ti-6Al-4V alloy (Figure 2b,c), as the large amount of open porosity means that there is plenty of N_2_ within the pore structure to be able to nitride the pore surfaces.

**Figure 4:**
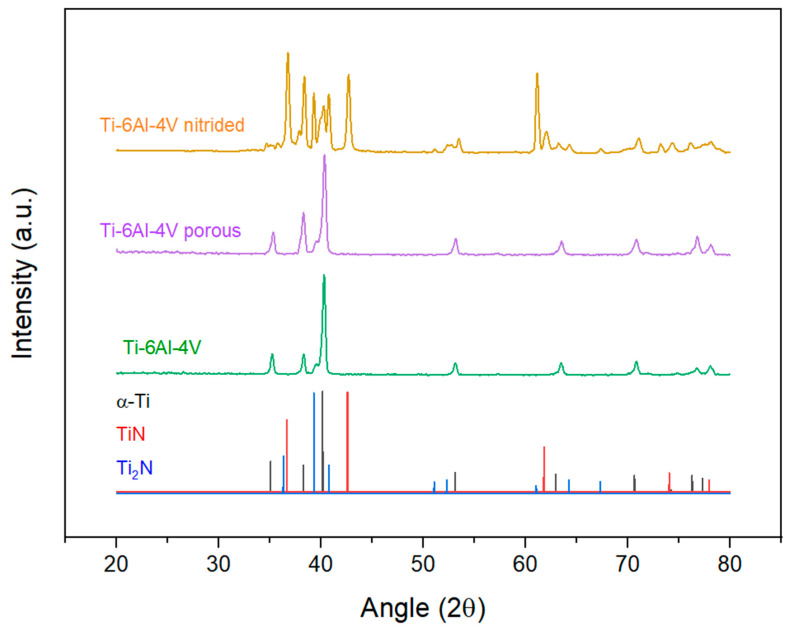
XRD patterns of as-sintered and nitride Ti64 samples.

The X-ray diffraction patterns are shown in Figure 4. In the case of the untreated Ti-6Al-4V, the pattern corresponds well with that of α-Ti, corroborated in the literature [41], with a low intensity peak next to the main peak corresponding to β-Ti, in the vicinity of 2θ = 40°. The amount of β-Ti is low, as also evidenced in Figure 2, so that the remaining peaks are not visible. There are no additional peaks appearing in the porous, untreated Ti-6Al-4V, whose main difference with the dense alloy is the addition of the space holder, ensuring that it has been completely removed and it has not affected the surface of the material.

**Figure 5:**
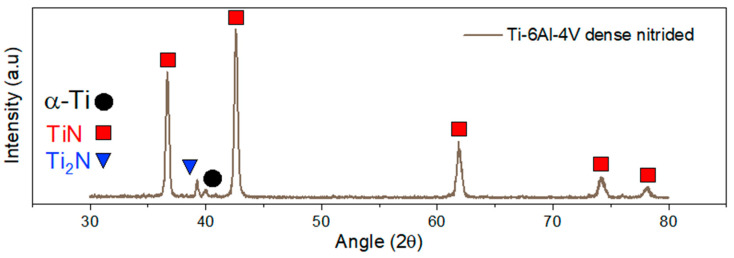
GIXRD patterns of nitride Ti64 samples.

The diffraction pattern of the nitrided Ti-6Al-4V alloy is more complex due to the formation of N-rich compounds, specifically TiN and Ti_2_N. It can be seen how the peak corresponding to β-Ti disappears, as the β phase is not stable with high N amounts and can be found only deeper than 5 µm, therefore being out of reach for the technique. The most intense signal corresponds to the (111) and (200) peaks of TiN [42]. The difference between the intensities of both nitride compounds is due to the thermal nitriding process itself, generating a diffusion gradient from the surface toward the interior of the sample. The phase richer in nitrogen, in this case TiN, was generated at the surface of the sample, while, deeper into the material, the nitrogen concentration decreases, forming a more nitrogen-depleted phase, Ti_2_N [43,44,45]. With GIXRD, we can determine the composition of the sample surface at an analysis depth of 0.75 microns. Looking at Figure 5, the most intense peaks correspond to the TiN phase, accompanied by less intense peaks corresponding to the Ti_2_N phase, which is in accordance with the layered structure mentioned above.

**Figure 6:**
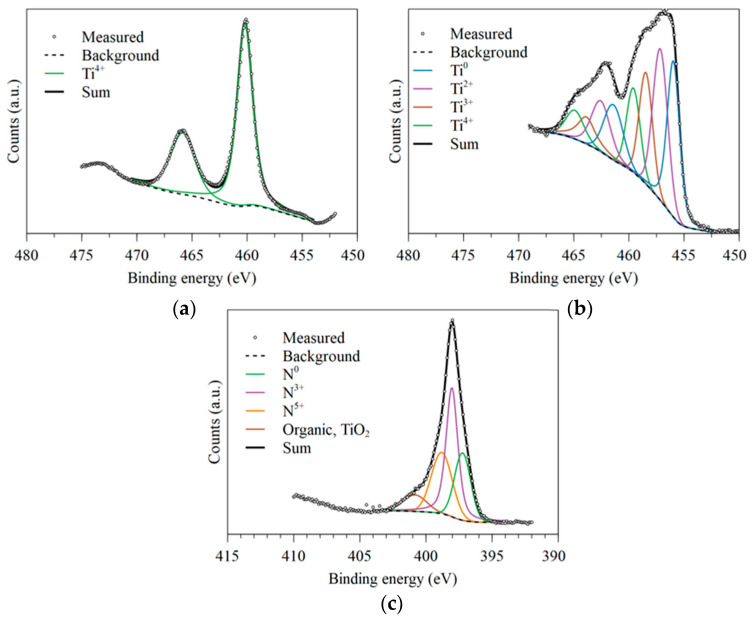
XPS spectra of Ti-6Al-4V: (**a**) Ti 2p of untreated Ti-6Al-4V, (**b**) Ti 2p of nitrided Ti-6Al-4V, and (**c**) N 1 s of nitrided Ti-6Al-4V.

For a more detailed study of the surface, XPS was carried out to characterize the composition of the sample semi-quantitatively. Figure 6 shows the spectra obtained for the Ti bands of both sintered and nitrided Ti-6Al-4V, as well as the nitrogen for the latter. The untreated Ti-6Al-4V (Figure 6a) has only a pair of peaks corresponding to the same 2p doublet, with a binding energy of around 460 eV for the 2p_1/2_ peak. This corresponds to Ti^4+^, a state associated with the TiO_2_ layer that spontaneously forms on the surface of titanium [43]. The Ti spectrum of the nitrided sample (Figure 6b) shows the contribution of the spectra corresponding to four different chemical species (0, +2, +3, +4), whose peaks corresponding to the 2p_1/2_ orbital are located at 456, 457, 458, and 460 eV, respectively. Following the literature [44,45], we can associate these peaks with the TiO_2_ (Ti^4+^), TiO_x_N_y_ (Ti^2+^ and Ti^3+^), and TiN (Ti^0^) phases. A semi-quantitative analysis of the XPS fitting for the distribution of the different states is shown in Table 2. In addition, the spectrum corresponding to the nitrogen analysis (Figure 6c) shows three distinct chemical states for N apart from those of organic N or N in solution within TiO_2_: one corresponding to the TiN phase and the other two corresponding to mixed oxynitrides, confirming the results seen in the Ti 2p spectra.

It is expected that, after nitriding, a thin layer of TiO_2_ forms naturally on the surface once in contact with the atmosphere, as this compound is more stable than TiN itself. It is likely that the oxynitride TiO_x_N_y_ is formed as an intermediate layer between the oxide and the nitride, while the TiN stays as the inner compound of the surface. As the penetration of the XPS instrumentation is around 10 nm, these results demonstrate that thermal nitriding develops a coating of TiN with layers of only a few nm of oxynitrides and oxides. Since the oxide and oxynitride layer formed is thin, the insulating effect of the oxide will be minimized, obtaining a surface with better electrical conductivity provided by TiN.

### 3.3. Corrosion Behavior

**Figure 7:**
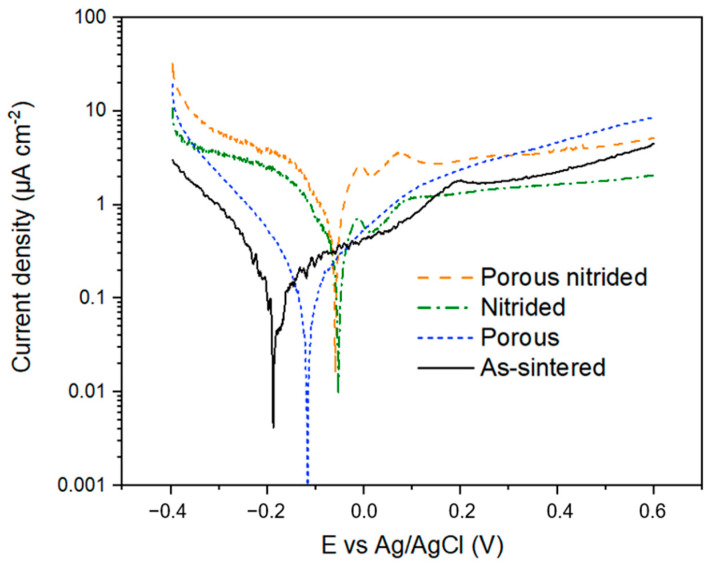
Potentiodynamic polarization curves of Ti-6Al-4V simulating the PEMFC anode conditions.

Figure 7 shows the behavior of the different Ti-6Al-4V conditions (untreated/treated and dense/porous) under a linear polarization test replicating anodic conditions for a PEMFC BP. The non-nitrided materials (as-sintered and porous) show a passive behavior, although slightly more active in the porous sample due to the larger surface area exposed to the medium, leading to higher corrosion rates of the whole sample. In the case of the nitrided samples, both show a behavior similar to active–passive. As the potential increases, the current density does not show a significant increase, remaining in the same range and reaching a dynamic equilibrium. Furthermore, it is observed that in the nitrided samples, the current density increases more slowly with increasing potential, probably due to the chemical reactions taking place on the surface of the sample. The occurrence of oxygen at the cathode of the PEMFC will increase the formation of the passive layer on the sample.

The corresponding electrochemical parameters, including corrosion potential (E_corr_), corrosion current density (i_corr_), and the anodic and cathodic Tafel slopes, are shown in Table 3. Additionally, the corrosion current density at 0.6 V vs. Ag/AgCl is shown, as it is used as a target to qualify materials according to the US DOE. The lowest current density at 0.6 V_Ag/AgCl_ is obtained for the dense nitrided sample, followed by the dense untreated sample. This indicates a higher corrosion in the porous samples due to the larger exposed surface; however, at 0.6 V_Ag/AgCl_ the current density of the untreated dense sample is similar to that of the porous nitrided sample, indicating the better performance provided by the titanium nitride layer adhered to the sample surface. Even though the current density obtained at 0.6 V vs. Ag/AgCl is higher than the target (1 μA·cm^2^), it is still within the same order of magnitude. Additionally, the corrosion mechanism of Ti in these pH and voltage conditions is growing a passive oxide layer instead of dissolving, hence being a safe option [7].

It is important to notice that the surface area used in the current density calculation for the porous samples is the geometrical surface area of the sample, not the surface area actually exposed to the electrolyte. Comparing materials with the same treatment, the actual corrosion rate should be similar, but the nominal corrosion rate should be much higher for the porous material. As the values are in the same current density range, bubbles could be forming within the porous sample, avoiding the wetting of the entire surface by the electrolyte. There is a potential for the development of differential aeration batteries along the porous structure as well, affecting how the porous material corrodes.

**Figure 8:**
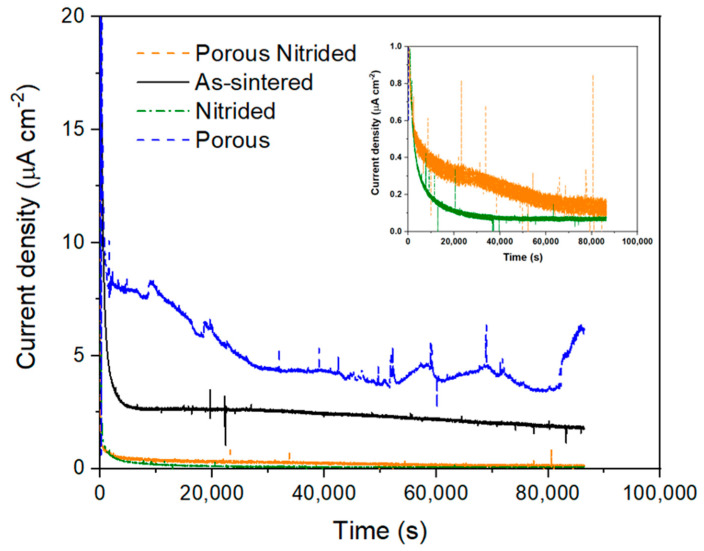
Chronoamperometric test of the Ti64 samples at 0.6 V vs. Ag/AgCl simulating the PEMFC cathode conditions.

To study the long-term stability in the most aggressive environment, 24 h tests at a constant potential were carried out simulating the cathodic conditions of a PEMFC. The curves of the different samples at +0.6 V_Ag/AgCl_ and in H_2_SO_4_ solution at pH 3 and air bubbling are shown in Figure 8. It can be seen how in the case of the samples that have not been nitrided, stationary values of more than 2 μA/cm^2^ are reached, higher than the target value of 1 μA/cm^2^. For the nitrided samples, values one order of magnitude lower with respect to the substrate are achieved, with the dense nitride sample again providing the lowest current density value, with a value of 0.08 μA/cm^2^. These values are within the target and demonstrate the importance of the surface coating in delaying the oxidation of the surface. Titanium nitride follows the corrosion mechanism indicated by the following reaction (Equation (6)) [46]:

(6) $$TiN+2\;H_{2}O\;↔TiO_{2}+\frac{1}{2}N_{2}\left(g\right)+4H^{+}+4e^{-}$$

However, layers corresponding to the presence of TiO_2_ and TiON have been observed in the XPS. Therefore, these layers will modify the corrosion mechanism of the specimen. Equation (6) can be divided into two stages: a first one, wherein TiN oxidizes to form TiON (Equation (7)) and a second stage, wherein TiON reacts to form TiO_2_ (Equation (8)) [12]:

(7) $$TiN+H_{2}O\;↔TiON+2H^{+}+2e^{-}$$

(8) $$TiON+\;H_{2}O\;↔TiO_{2}+\frac{1}{2}N_{2}\left(g\right)+2H^{+}+2e^{-}$$

Observing how the intensity remains constant during the tests of the nitrided samples, it can be assumed that the kinetics of Equation (8) are higher than those of Equation (7), decreasing the corrosion rate of the nitrided layer. Therefore, corrosion is due to the increase in the surface oxide layer.

These reactions rule the corrosion mechanisms, assuming that the dissolution of Ti^4+^ is less prone to happen because of the diffusion required through the different surface layers, explaining the different behavior observed between the as-sintered and the nitrided samples.

### 3.4. Interfacial Contact Resistance

**Figure 9:**
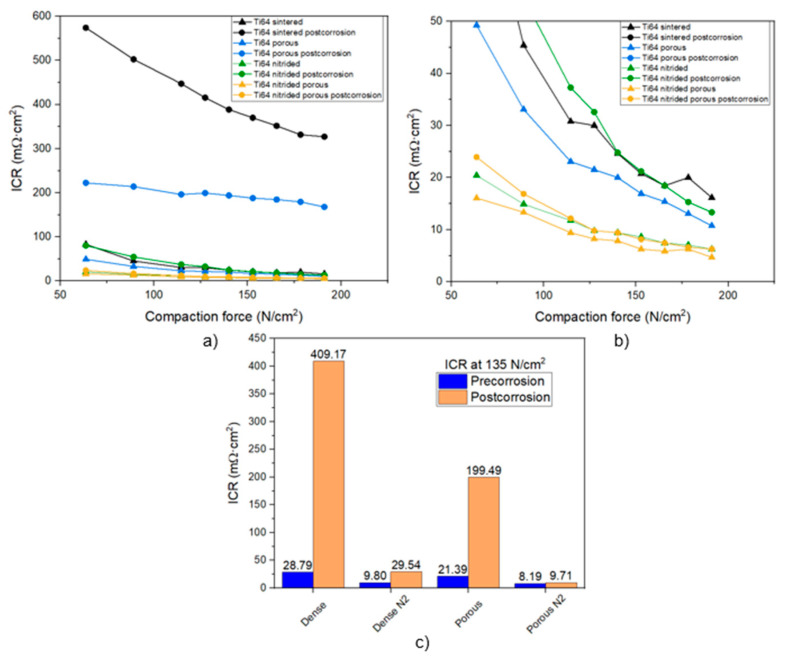
Interfacial contact resistance (ICR) of untreated and treated Ti-6Al-4V: (**a**) evolution with compaction force, (**b**) insert of (**a**) centered on the low ICR values, (**c**) comparison of pre- and post-corrosion measured at 135 N/cm^2^.

The interfacial contact resistance (ICR) of the different Ti-6Al-4V alloys is shown in Figure 9, which includes the ICR of the material in the pristine condition and also after corrosion testing. The non-nitrided samples show higher ICR values due to the TiO_2_ layer formed on the surface. In the pristine condition, the untreated Ti-6Al-4V samples have ICRs ranging around 20 mΩ·cm^2^ in compaction pressures in the range of the application, which is higher than the DOE target but still within range. These values mean that, before corrosion, the insulating oxide layer must be relatively thin. The deposition of a TiN layer on the surface provides a considerable improvement, decreasing the ICR below 10 mΩ·cm^2^, which meets the technical requirements of DOE 2020. The oxide and oxynitride layers formed in the outer surface of the material are only a few nm thick, and the ICR is controlled by the good conductivity of TiN.

The improvement is significantly more remarkable when analyzing the ICR of the samples after corrosion. It is observed that the increase in ICR is much higher in the non-nitrided samples due to the fast growth of the TiO_2_ layer on the surface associated with the higher corrosion rate of Figure 7. However, in the nitrided samples, this increase is much lower, because the presence of TiN causes a very slow formation of TiO_2_ and TiO_x_N_y_ on the surface during corrosion. Therefore, it is able to maintain a high ICR from the better electrical conductivity provided by the nitride. In this way, the nitrided porous sample maintains ICR values below 10 mΩ·cm^2^, meeting the DOE objectives.

It is important to highlight the role that porosity plays in the ICR measurement. Figure 9c shows how, in the pre-corrosion measurements, the ICR values obtained are similar between the nitrided and non-nitrided samples. Taking into account that the values have been obtained using the geometrical surface and not the real surface, the ICR values in the porous samples should be lower than those shown. However, the deformation of the GDL due to the pressure applied during the test has to be taken into account, generating GDL/sample contact at the edges of the pores and increasing the effective surface area.

The biggest difference observable between the samples is when comparing the post-corrosion values between the dense and porous samples. In contrast to the dense samples, where the TiO_2_ passive layer is more homogeneous, in the porous samples, the TiO_2_ layer is more irregular on the surface, presenting variable thickness. This heterogeneity in the layer allows maintaining a lower ICR after corrosion, fulfilling the technical requirements.

## 4. Conclusions

The aim of this study was to develop and characterize porous Ti-6Al-4V structures with surface modification using powder metallurgy as a cost-effective way to obtain alternative designs for use as bipolar plates for PEMFC.

- Structures with a controlled porosity size and volume fraction can be obtained using the space-holder technique. The use of space holders provides an open and interconnected porosity, and the presence of porosity from the sintering process creates a bimodal distribution.
- Surface modification by thermal nitriding provides a multilayer coating consisting of TiN as the external layer and Ti_2_N as the internal layer. Thicknesses of several thousand nanometers are obtained for both dense and porous materials. However, the thickness is heterogeneous within the pores.
- Within the first tens of nm of depth of the surface, there is the presence of Ti oxides and oxynitrides, which form naturally. However, their presence does not hinder the nitrided alloys from reaching the targeted values of ICR.
- The treatment leads to an improvement in the ICR of the nitrided samples, obtaining values below 10 mΩ·cm^2^, 9.8 mΩ·cm^2^ for the dense sample and 8.2 mΩ·cm^2^, meeting the requirements.
- Corrosion resistance is also improved by the addition of the TiN coating; owing to the corrosion mechanism generated by the presence of the nitride, stable and thin oxide layers are obtained that improve the passive behavior of the structure, allowing low ICR values to be maintained after the corrosion tests, especially in the porous samples. For cathodic conditions the target values are met, but in the case of anodic conditions, the nitrided samples improve the corrosion behavior but do not meet the requirements, even though they are in the same order of magnitude. Regardless, the corrosion mechanism allows the material to be used safely, as there is no dissolution of species that can affect the active materials of the PEMFC.

## Figures and Tables

**Table 1:** Porosity of the dense and porous samples of Ti64.

Sample	Open Porosity (%)	Closed Porosity (%)	Total Porosity (%)
Ti-6Al-4V dense	4.73	7.92	11.71
Ti-6Al-4V porous	41.67	10.23	51.90

**Table 2:** Fitting results of the Ti 2p XPS spectra.

Sample	Ti^0^ (at%)	Ti^+2^ (at%)	Ti^+3^ (at%)	Ti^+4^ (at%)
As-sintered	0	0	0	100
Nitrided	31.38	31.44	20.82	16.36

**Table 3:** Electrochemical parameters of the polarization curves for the anode side.

Specimen	E_corr_ (V)	i_corr_ (μA/cm^2^)	βc (V/dec)	βa (V/dec)	i at + 0.6 V vs. Ag/AgCl (μA/cm^2^)
As-sintered	−0.188	0.154	0.217	0.283	4.50
Porous	−0.117	0.156	0.208	0.207	8.61
Nitrided	−0.053	0.423	0.180	0.430	2.08
Porous nitrided	−0.056	1.547	0.267	0.298	5.15

## Data Availability

The data presented in this study are available on request from the corresponding author. The data are not publicly available due to privacy.
